# P-664. Trends in Bacterial Pneumonia-Related Mortality Among Older Adults in the United States

**DOI:** 10.1093/ofid/ofae631.861

**Published:** 2025-01-29

**Authors:** M Danial Ali Shah, Sadaf Aslam, Beata Casanas

**Affiliations:** King Edward Medical University, Lahore, Punjab, Pakistan; University of South Florida Morsani College of Medicine, Tampa, Florida; University of South Florida, Tampa, Florida

## Abstract

**Background:**

Bacterial pneumonia is one of the leading causes of mortality in the US, particularly among those aged 65 and older. No study has been done on this population to understand mortality patterns based on demographic characteristics such as age, gender, race, and geographic regions, which helps to enhance evidence-based interventions for this vulnerable age group.

**Figure:**
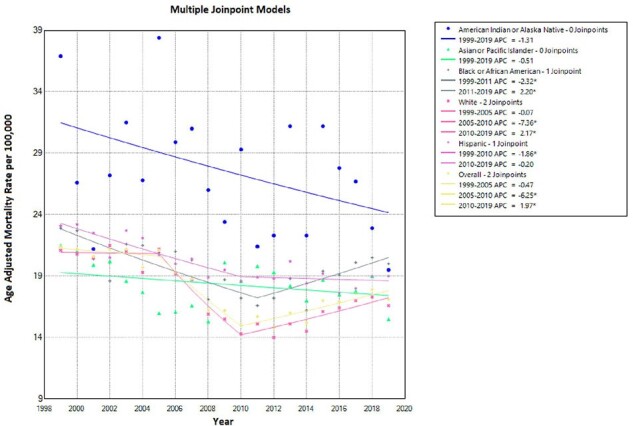

Trends in Bacterial Pneumonia-related AAMRs and APC stratified by race in older adults.

**Methods:**

This study involves a two-decade CDC WONDER data on bacterial pneumonia-related deaths via ICD-10 codes A48.1 and J13-J16.0 in ≥ 65-year-olds from 1999-2019. Demographic disparities are analyzed based on age, gender, race, census region, and urban-rural classification using systematic database analysis. Age-adjusted mortality rates (AAMRs) per 100,000 and annual percent change (APC) with a 95% Confidence Interval (CI) are calculated through joinpoint regression analysis.

**Figure:**
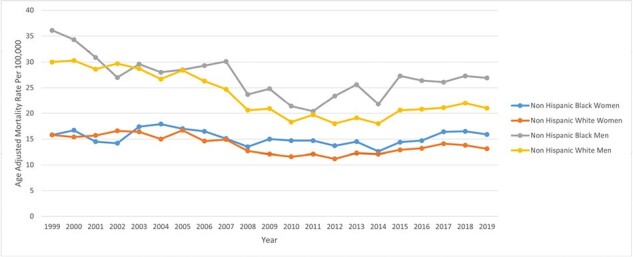

Trends in Bacterial Pneumonia-related AAMRs in Black and White men and women.

**Results:**

155,819 individuals aged ≥65 died from bacterial pneumonia in the US from 1999 to 2019. Initially, AAMR declined (1999-2005: APC -0.47; 95% CI -1.53 to 1.08), followed by a steep decline (2005-2010: APC -6.25; 95% CI -9.72 to -4.51), and concluded with a significant increase (2010-2019: APC 1.97; 95% CI 1.09 to 3.12). Males had higher AAMR (23.6) than females (14.2). Hispanics (19.6) were more affected than non-Hispanics (17.8) and Blacks more than Whites, except for White men (23), surpassing Black women (15.3). Pneumonia mortality varied by region, highest in Arizona and California (44.5 and 30.4) and lowest in New Jersey and Wisconsin (8 and 9.4). Nationwide, Black/White mortality is comparable except for the Northeast, where Black mortality doubles that of Whites.

**Figure:**
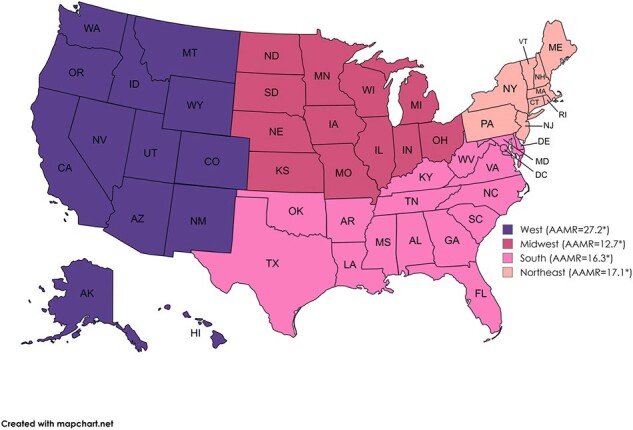

Trends in Bacterial pneumonia-related AAMRs stratified by census regions. *=significant at p < 0.05; confidence interval does not include zero.

**Conclusion:**

While initial reductions in bacterial pneumonia-related mortality among older U.S. adults were observed, an upward trend from 2010 to 2019 negated this progress, resulting in a concerning resurgence. This upsurge disproportionately impacts specific demographics, including those aged 85 and older, males, non-Hispanic Blacks, those in the Western U.S., and urban populations. To further reduce mortality rates, this highlights the urgent need for effective interventions, improved access to care, and age-specific preventive programs. Further research is required to understand contributing factors.

**Figure:**
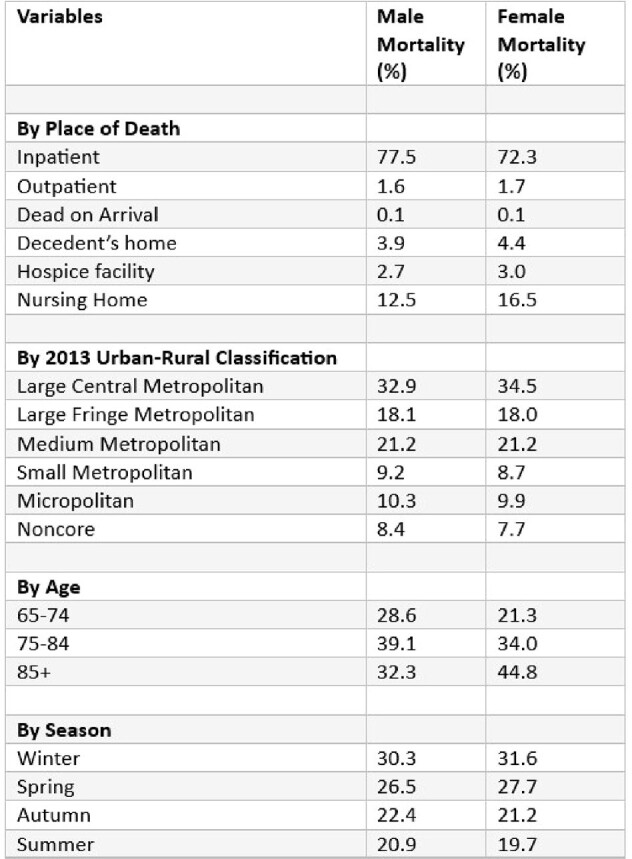

Bacterial Pneumonia-related crude mortality percentage stratified by different variables between men and women.

**Disclosures:**

**All Authors**: No reported disclosures

